# Current situations and future directions for mental health system governance in Nepal: findings from a qualitative study

**DOI:** 10.1186/s13033-017-0145-3

**Published:** 2017-06-08

**Authors:** Nawaraj Upadhaya, Mark J. D. Jordans, Ruja Pokhrel, Dristy Gurung, Ramesh P. Adhikari, Inge Petersen, Ivan H. Komproe

**Affiliations:** 1 Transcultural Psychosocial Organization Nepal, Kathmandu, Nepal; 2 Department of Research and Development, HealthNet TPO, Amsterdam, The Netherlands; 3 Centre for Global Mental Health, Institute of Psychiatry, Psychology and Neuroscience, King’s College London, London, UK; 4 School of Nursing and Public Health Medicine, University of KwaZulu-Natal, Durban, South Africa; 5 Utrecht University, Utrecht, The Netherlands

**Keywords:** Governance, Mental health services, Mental health system, Global mental health, Nepal, Low- and middle-income countries

## Abstract

**Background:**

Assessing and understanding health systems governance is crucial to ensure accountability and transparency, and to improve the performance of mental health systems. There is a lack of systematic procedures to assess governance in mental health systems at a country level. The aim of this study was to appraise mental health systems governance in Nepal, with the view to making recommendations for improvements.

**Methods:**

In-depth individual interviews were conducted with national-level policymakers (n = 17) and district-level planners (n = 11). The interview checklist was developed using an existing health systems governance framework developed by Siddiqi and colleagues as a guide. Data analysis was done with NVivo 10, using the procedure of framework analysis.

**Results:**

The mental health systems governance assessment reveals a few enabling factors and many barriers. Factors enabling good governance include availability of mental health policy, inclusion of mental health in other general health policies and plans, increasing presence of Non-Governmental Organizations (NGOs) and service user organizations in policy forums, and implementation of a few mental health projects through government-NGO collaborations. Legal and policy barriers include the failure to officially revise or fully implement the mental health policy of 1996, the existence of legislation and several laws that have discriminatory provisions for people with mental illness, and lack of a mental health act and associated regulations to protect against this. Other barriers include lack of a mental health unit within the Ministry of Health, absence of district-level mental health planning, inadequate mental health record-keeping systems, inequitable allocation of funding for mental health, very few health workers trained in mental health, and the lack of availability of psychotropic drugs at the primary health care level.

**Conclusions:**

In the last few years, some positive developments have emerged in terms of policy recognition for mental health, as well as the increased presence of NGOs, increased presence of service users or caregivers in mental health governance, albeit restricted to only some of its domains. However, the improvements at the policy level have not been translated into implementation due to lack of strong leadership and governance mechanisms.

## Background

Governance is a key determinant for economic and social advancement and overall health systems development [1]. As different parts of health systems interact with each other, it is evident that assessing and understanding governance are crucial to improve the performance of health systems [2]. WHO’s 2000 World Health Report describes governance as ‘stewardship’, which was defined as “*setting and enforcing the rules of the game and providing strategic direction for all the different actors involved*” [3]. In 2007, ‘governance and leadership’ was included as one of six building blocks of a health system (others included the health workforce, health financing, service delivery, information management and medical products and technology), and was defined as “*ensuring strategic policy frameworks exist and are combined with effective oversight, coalition building, the provision of appropriate regulations and incentives, attention to system design and accountability*” [4].

In the last decade, health governance has received significant academic attention and has been recognized as a critical element of the health systems strengthening agenda [5]. However, globally, governance for mental health systems still remains conceptually underdeveloped despite initiatives taken to improve clinical governance of mental health through an integrated organization-wide (institutional) approach for continuous quality improvement [6]. This issue of mental health systems governance warrants further attention, given that mental health problems account for about 12% of global burden of disease [7].

The little progress that has been made in clinical governance of mental health is associated with the limited capacity to the monitoring and standardization of drug prescription practices [8], leaving out many other aspects of mental health systems governance which play vital roles for strengthening mental health systems. Effective and efficient mental health systems governance is one of the strategies to deal with the high burden of mental illness in low- and middle-income countries. In Nepal, although there is no nationally representative data on disease burden, it can be assumed that, due to lack of resources, expertise and overall governance, both the prevalence and burden of disease for mental health is higher than the global figure. The small-scale studies available have indicated that around 20–25% of all patients visiting primary health care facilities have shown psychiatric morbidity [9], while suicide accounted for 16% of deaths among women of reproductive age [10].

It is increasingly recognized that, in order to achieve the intended results in the overall development of health systems, not only the resources but also the governance and accountability mechanisms need to be in place [11]. The roles of the Ministry of Health, service providers and service users need to be taken into account [12]; for that, governance processes and accountability mechanisms that are responsible for the overall development of the health systems need to be implemented and assessed periodically to identify and address system-level barriers. To do this, better understanding of mental health needs, service availability and utilization, governance procedures and mechanisms, and the interaction between these variables is essential. There are a few studies in Nepal that have touched upon some themes of mental health systems such as policy and legislative frameworks [13, 14], but their focus was mainly on mental health service delivery rather than on mental health governance. Since 2013 mental health systems related studies are conducted as part of the Emerging Mental Health Systems in Low- and Middle-Income Countries (EMERALD) research program which aims to support mental health systems strengthening in six countries (Ethiopia, India, Nepal, Nigeria, South Africa and Uganda) [15].

In this paper, we used Siddiqi and colleagues’ health system governance assessment framework to report on systems-level constraints and facilitating factors because it provides a set of questions that cover each governance principle at the national, policy formulation and implementation level. This framework has been previously applied in Pakistan, where there are similar health system barriers and opportunities compared to Nepal [1]. The ten governance principles of Siddiqi and colleagues’ framework and the themes included in this study are presented in Table 1.

**Table 1:** Governance principles and associated themes. Adopted from Siddiqi et al. [1]

Governance principles	Broader themes included in the study
Strategic vision	Facilitative factors and barriers to development and implementation of plans and policies
Participation and consensus orientation	Facilitative factors and barriers to coordination and consultation with service providers, service users and other sectors outside of health
Rule of law	Facilitative factors and barriers to the development and enforcement of laws, as well as synergy between laws
Transparency	Facilitating factors and barriers to ensuring transparency in resources allocation, decision making, appointment and transfer of staff
Responsiveness and integration of care	Facilitating factors and barriers to integration of mental health in the health facility as well as in the community. Burden of mental illness, priority given to mental health
Equity and inclusiveness	Facilitating factors and barriers to mental health financing, access to services and anti-stigma programs
Effectiveness and efficiency	Facilitating factors and barriers to human resources capacity building, mental health infrastructure development and supply chain management of psychotropic drugs
Accountability	Facilitating factors and barriers to ensuring effective enforcement of accountability measures. The role of press, elected bodies and judiciary in ensuring accountability
Intelligence and information	Facilitating factors and barriers to mental health data recording, reporting, analysis and dissemination
Ethics	Facilitating factors and barriers to service user satisfaction and quality assurance, as well as mechanisms for safeguards against unethical research

### The Nepalese health governance context

The unstable political history of Nepal (internal conflicts among the rulers, several political movements against the government and frequent changes of government during the Rana Regime, multiparty democracy, and Maoist insurgency) has resulted in overall poor governance but has particularly affected governance in the health sector. At the same time, there exist quite a few laws and policies aimed at strengthening governance in the health sector. For example, the 2006 Interim Constitution of Nepal, promotes a more decentralized system of governance and guarantees the rights to equality of all citizens regardless of their social, cultural or economic background and physical or mental health status [16]. Similarly, the Local Self-Governance Act of 1999 gives authority to the local bodies to operate and manage health institutions at local level [17]. Following this, the Ministry of Health handed over 1433 health institutions to local health management committees, expecting changes in terms of decision-making power structure and accountability mechanisms. However, no noticeable changes have been observed, and the health system could not ensure the needs and utilization of health services, nor could it involve local people in decisions which have an impact on the health situation of the local community [11].

## Methods

### Setting

This study was carried out at two sites in Nepal: (1) the Chitwan district, which included district-level health managers and planners, and (2) Kathmandu, the capital city, which included national-level policymakers and planners. Because context has an effect on governance, these sites were chosen to compare the national urban setting (Kathmandu) with a district rural setting (Chitwan). The Chitwan district has a population of almost 580,000 and is a regional center for business and medical services. Hospital-based mental health services are being provided by government as well as private hospitals based at district headquarters. Mental health services in the community were non-existent until 2011; since then, a pilot community mental health program has been implemented in the health facilities of Chitwan through government-NGO collaboration [18].

Kathmandu city was selected to include national-level policymakers and planners working in the Ministry of Health, Department of Health Services, government hospitals and departments providing mental health services. In Kathmandu valley, both medical and psychosocial support is available through government as well as private sectors. Some NGOs and private groups provide residential treatment services for people with mental illness.

### Sampling

The sampling of respondents for the study was divided into two broad categories namely (a) district-level health care managers and planners, and (b) national-level policymakers. Purposive sampling was used to identify key informants based on their current role and position in mental health care policy development and management, and potential influence for future policy development. Based on the study team’s knowledge, possible respondents were identified and approached. At the district level, health staff involved in mental health programs at the District Public Health Office (DPHO), district hospitals and primary health care facilities were approached for an interview. The selected district level participants (n = 11) included health managers such as medical superintendents, health assistants, public health officers and public health inspectors. At the national level, the research team decided to select participants from various professional backgrounds (clinical, human rights, law enforcement, health policy and planning, psychology and mental health research) relevant to mental health policy formulation. The selected national-level participants (n = 17) included: psychiatrists, psychologists, public health officers, under-secretaries from ministries, primary health care in-charges, human rights workers, a police officer, and a research officer who were involved in mental health policy and planning. Table 2 provides the characteristics of the respondents.

**Table 2:** Characteristics of respondents

Characteristics	Respondents n (%)
Gender	
Male	23 (82.14)
Female	5 (17.85)
Total	28 (100)
Profession	
Psychiatrist	4 (14.28)
Human rights worker	4 (14.28)
Public health officer	8 (28.57)
Psychologist	2 (7.14)
Ministry representative	4 (14.28)
Primary health care in-charge	4 (14.28)
Researcher	1 (3.57)
Police officer	1 (3.57)
Total	28 (100)

### Procedures

A semi-structured interview schedule was developed guided by Siddiqi and colleagues’ health system governance framework [1] and adapted to the Nepalese context. For the contextualization process, first the English questionnaire was translated to Nepali by two academic researchers. Then each question of the draft translation was discussed among the research team to determine whether or not the translation captured the real meaning of the question. Once the Nepali textual translation was agreed (consensus), an interview role-play on each question was done to explore the type of responses the question would generate. The validity of the translation was evaluated on two criteria: would the responses make sense in the study locations and were the responses valid for assessing the specific area of the governance framework.

The major areas covered by the interview schedule were: (a) service planning and management for mental health care integration, (b) human resources, (c) equipment and infrastructure for mental health care integration, (d) capacity-building needs, (e) laws and regulations pertaining to mental health, (f) funding, (g) monitoring of mental health policies and services, and (h) quality assurance and ethics.

A team of six experienced researchers (university-level education and average 3 years of research experience) collected data between March and June, 2014. All of them had participated in the translation and validation of the interview checklist and received a 1-week training on qualitative research methods, prior to data collection. In total, 28 interviews (of approximately 45 min to 1 h long) were conducted by visiting the participants at their work places. The interviews were audio-taped, transcribed and then translated into English by bilingual translators. The researchers made check for accuracy of this translation. As many participants made reference to national policies and provisions related to governance but did not know the details of such provisions, additional data was collected by reviewing relevant national-level policy documents. The findings of the document review were charted as per the governance principles and, where relevant, used to substantiate the arguments made by the participants.

### Data analysis

Given the descriptive and exploratory nature of the study, we adopted a qualitative research methodology informed by a framework method of data management and analysis [19]. The five stages of framework analysis (familiarization, identifying a thematic framework, indexing, charting and mapping, and interpretation [20]) suited this study as the approach makes use of the priori themes identified by Siddiqi and colleagues’ health governance framework.

A random selection of the transcripts were read separately by two researchers (DG and RP) who then generated broad themes and codes within the themes. To incorporate alternative viewpoints, the two sets of ‘open codes’ developed by the researchers were shared for ‘peer-concept’ validation with members of the EMERALD project who were involved in research design and data collection. Based on discussion of the preliminary framework, adaptations were made to develop a final analytical framework keeping the categories of Siddiqi and colleagues’ framework as parent themes [1]. The transcripts were uploaded in qualitative data analysis software, NVivo-10. The analytical framework was applied to all the uploaded transcripts and, during the coding process, it was further refined. Using the framework matrix option in NVivo, the data were charted and summarized. The framework matrices were exported from NVivo to an Excel Spreadsheet and cross-checked by the researchers who were involved in data collection. When inconsistencies were noted, they were verified with original transcripts. The selection of text under each parent theme mentioned in the results section is based on the data derived from this procedure (Table 3).

**Table 3:** Mental health systems governance matrix for Nepal

Governance principles	Summary of challenges and enabling factors			Recommendations
	National level	Health policy formulation level	Policy implementation level	
Strategic vision	Interim constitution 2006 recognizes health as a fundamental right	National mental health policy 1996 exists; no revisions to date	Lack of implementation of 1996 mental health policy	Integrate mental health policy provisions in 5 year health plan; establish an accountable mental health unit in MoH
Participation and consensus orientation	Lack of participation of civil society, private sector and government departments in mental health decision making	Increasing consultation with NGOs and other stakeholders in mental health policy formulation	In some districts, the District Public Health Office and NGOs are partnering to provide mental health services in primary health care centers	Establish coordination and collaboration within and beyond health sectors; build consensus among stakeholders regarding involvement of service users in policy, planning and service delivery
Rule of law	Consumer Protection Act 1998 bans the production and sale of goods which are harmful to consumers’ health	Fourth revisions of draft mental health act, but no official endorsement	Mechanisms to monitor misuse of psychotropic drugs in place but rarely implemented	Ratify mental health legislation in line with national and international human rights laws and legislation, and strictly enforce legal provisions in practice
Transparency	Ranked 126 out of 175 by Transparency International on corruption perception index	Processes and mechanism for ensuring transparency in mental health resource allocation and expenditure not well defined	Performance assessment, promotion and transfer of district health manager and district health staff differ in policy and practice	Develop mechanisms and procedures for monitoring and supervision of mental health programs to ensure transparency in health sector
Responsiveness to patients’ health needs	The government policy and programs (2014) state the provision for national insurance program	Health Policy 2014 recognizes the need to increase state’s investment to cover all health care expenses of disabled people, including psychosocial disability	In the absence of clear policy guidelines to address responsiveness, the medical and non-medical expectations of populations not met	Arrange motivational incentives and facilities for health worker retention in rural areas; strengthen mental health information use for the allocation of budget
Equity and inclusiveness	Social security allowance is given to elderly, widow and disabled, including psychosocial disability	Nepal Health Policy 2014 mentions that health services will be provided to poor, marginalized and vulnerable population on the principles of equity and social justice	Knowledge about existence of disability allowances for people with mental illness is low, so very few people have received allowances	Disseminate information about services available in the community for treatment of mental illness and benefits provided by the government
Effectiveness and efficiency	Limited coordination, communication and consultation between bureaucrats and technocrats (clinicians)	Frequent turnover of policymakers; no mental health unit under MoH for policy and planning	Other support systems, such as supply of psychotropic drugs and regular supervision and monitoring, are lacking	Regulate policy on staff turnover; provide training to health workers; ensure regular supply of essential psychotropic drugs and arrange separate counselling room within primary health care facilities
Accountability	Public accounts committee of the parliament looks after accountability	Mechanisms and processes for overseeing financial and administrative adherence in place	No evidence on the effective enforcement of accountability measures	Implement existing mechanisms and processes to make the health system accountable to the population in need of mental health services
Intelligence and information	No national level study on mental health conducted	The HMIS data not used in mental health policy, planning and service provision	Mental health data collected from Out Patients Department registers maintained in district health facilities	Build capacity of government staff for systematic record keeping, and monitoring and evaluation of mental health programs
Ethics	Ethics of mental health research is monitored by Nepal Health Research Council	To ensure bioethics, Nepal Health Research Council has an ethical review committee, and a monitoring and evaluation section	Nepal Health Research Council monitoring team makes field visits to oversee the research activities of the organizations	Sensitize researchers and health workers towards more ethical practice in the field of mental health research and service delivery

## Results

### Strategic vision and rule of law

Some respondents had the opinion that existing policies and plans related to mental health were not implemented in practice due to lack of leadership and infrastructure. The example provided by some of these respondents included the mental health policy of 1996 and the Nepal Health Sector Program II (NHSP II), which recommended the integration of mental health into primary health care. However, in almost all districts of Nepal, mental health services are not available from primary health care centers; even district hospitals in large parts of Nepal do not have mental health services. A national-level policymaker said,

“*If you see Nepal Health Sector Program II (NHSP II), we have kept Mental Health as an important pillar in the Non*-*Communicable Disease chapter thinking that it is important component…..but we have not been able to move forward in this process as we had to….*” (National Representative 02, Male).

This awareness of the non-implementation of NHSP II was particularly mentioned in relation to the failure to include mental health services within the Essential Health Care Services (EHCS) package, and piloting and scaling up of community-based mental health care. Likewise, a few respondents reported that NHSP II had the provision to appoint a focal point for mental health within the Ministry of Health (MoH), but no such focal point currently exists. Similarly, most of the national-level respondents mentioned that the non-communicable disease Multi-sectoral Action Plan (2014–2020) had the provision for the establishment of a mental health unit, but this also does not yet exist.

Some respondents mentioned that, in the absence of an official mental health act (despite the draft act being revised four times), many people with mental illness are suffering from the discriminatory provisions in other laws and legislation. A respondent from national level said:

“*There still exists discriminatory provisions in existing laws such as Civil Code of Conduct, NGOs registration act. The discriminatory laws related to mental health should be removed. State should be playing the role of guardian and ensure the right of all the citizens including people living with mental illness*” (National Policymaker 11, Male).

Most of the respondents found the government was not committed to mental health and, as a result, the situation has not much improved since 1996:

“*No matter how much it* [government] *says that it isn’t* [the situation of mental health services]*, evidence shows that in 20* *years it is still similar. Another thing is, generally, mental health problems are not visible. In our Nepal, they say in the villages that ‘Ban dadheko sabaile dekcha, maan dadheko kosaile dekhdaina.’* [Everyone will find out if the forest is burning but no one will find out if a heart is burning]” (National Policymaker13, Male).

Most respondents recognized this scenario for mental health as this has not been prioritized in Nepal. For example, respondents reported that there is no functioning unit or regulating mechanism of mental health in the MoH. Some respondents with a human rights background recommended revising the existing policy documents to include human rights perspectives and give priority to all forms of disability, including mental illness, so that mental health services are accessible to all, without discrimination. They stressed the importance of ‘equality’ in providing treatment and suggested policy provision for a human rights monitor to assess the situation of mental health services throughout Nepal. The policy provisions for psychosocial care for people with mental illness, and focus for gender issues and child mental health, were recommended by all the respondents.

### Transparency and accountability

Compared to other governance principles, transparency and accountability were much less discussed by the respondents. They either did not have information on these themes or they did not want to share this. The district-level policymakers did not clearly respond to questions regarding transparency of budget and decision making, and there were mixed responses from the national-level policymakers. A few national-level policymakers thought that there is transparency of the mental health budget since budgeting is done through participatory sharing and discussions, budgets are audited every year, and money is spent as directed by the law. However, other participants were of the view that the mental health budget was not transparent since it has not been allocated systematically and there is no public information on the budgetary decisions.

Some respondents who talked about transparency referred to it mainly in terms of budget allocation, budget expenditure and human resources management. A participant said,

“*Since there is no clear budget allocation for mental health, it cannot be said that the programs and budget are transparent.*” (National Representative 05, Male).

In terms of accountability, this was defined by the participants as responsiveness on the part of policymakers and health care managers to meet the mental health needs of the population.

### Monitoring of mental health services and policies

According to some respondents, there was no monitoring conducted and there was a lack of clarity as to who is responsible for this function. Most national-level respondents thought that the MoH was the responsible body for monitoring mental health policies and services. Others thought that monitoring was the duty of the Department of Health Services (DoHS). Some respondents identified a lack of qualified human resources, clarity of roles and tools for monitoring as some of the barriers to effective monitoring:

“*We haven’t been able to monitor all the organizations fully. This is one weakness. We aren’t able to go to the field and do the monitoring. We are working on improving this part.*” (National Representative 15, Female).

Some of the respondents were of the opinion that if these monitoring and evaluation (M&E) structures could be oriented towards mental health, the monitoring of mental health services could be easily implemented on a regular basis. Respondents also identified factors that could enable monitoring of mental health services in the existing health care system. For example,

“*Public Health Administration, Monitoring Evaluation Division under Ministry of Health monitors overall health system*” and ‘*Integrated Supervision Program’ does the overall monitoring and supervision of all health services in general.*” (National Representative 05, Male).

### Responsiveness and integration of care

In some respondents’ views, both responsiveness to the mental health needs of the population and integration of mental health in primary health care were ignored by the government. There is only one mental hospital at the central level, and respondents found that this was insufficient to cater for the needs of the country. One of the facilitating factors for responsiveness was reported to be the existence of the Good Governance and Management Act 2008, which has mechanisms redressing grievances at the national, regional, zonal and district level. However, some respondents were of the opinion that such mechanisms had not been practiced in the case of mental health. One national-level policymaker said:

“*During the monitoring, no such type of mechanism was seen which listened to the complaints or grievances regarding mental health…*” (National Representative 11, Male).

In the absence of proper monitoring mechanisms, some respondents found that the health facilities have not been accountable or responsive to the mental health needs of the population.

### Participation, coordination and collaboration

#### Involvement in policy and planning

Respondents reported that, in recent years, the participation in mental health policy and planning of various stakeholders from NGOs, the private sector and the service user community has improved. MoH has been formulating draft policies related to mental health through consultation workshops and meetings. However, some of the respondents expressed that the participation of service users, their caregivers and district-level service providers in policy and planning remained limited.

There were diverging views and opinions about service users’ involvement in policymaking. Some respondents found service user and caregiver involvement to be essential. For example,

“*Without their involvement, policymaking would be incomplete.*” (District Representative 09, Male), “*Since they are the one[s] who are facing the problem, their input is a must*” (District Representative 01, Male), and “‘*Nothing about us, without us’. So I think participation will come as a first condition.*” (National Representative 11, Male).

Among those who stressed the need for service user involvement, some said that there should be criteria for selecting service users such that only those individuals who can contribute in policy and planning should be consulted.

There were also views that policymaking was not an ideal platform for service user or caregiver involvement. Some respondents thought that mental health policy development is the domain of mental health experts where service users have no role. A respondent mentioned,

“*If we plan to involve them at the initial phase of policy making, then it would sound like, ‘Tauko le Puchar hallaune ki puchar le tauko hallaune’* [Whether head shakes the tail or tail shakes the head - it is always the head that shakes the tail, so that involvement of service users in policy and planning seem irrelevant]” (National Representative 09, Male).

### Involvement in service delivery

Some district-level respondents found that involvement of other government institutions and NGOs in service delivery has improved, especially in programs run by NGOs in collaboration with government agencies:

“*Service is delivered in consultation with sectors like VDC* [Village Development Committee]*, police or teacher.*” (District Representative 09, Male).

However, coordination and collaboration with stakeholders in service delivery and service integration was thought to be insufficient by some of the district-level respondents. They suggested greater coordination and collaboration among schools, VDCs, NGOs and health facilities for integration of mental health services into primary health care.

### Effectiveness and efficiency

#### Human resources capacity

Some respondents identified limited training of health workers in mental health and frequent transfers of health staff trained in mental health as two major problems in human resources for mental health. According to the respondents, the changes in the national government have brought about high staff turnover at district level, even before completing the mandatory 2 years of service in a place.

Lack of awareness and knowledge about mental health issues among health workers was seen by the respondents as another barrier. The respondents acknowledged that most health workers lacked this knowledge because their pre-service training did not cover mental health and even if it did, due to lack of practice, the health workers had mostly forgotten what had they learnt. One strategy suggested by several of the respondents to overcome this problem was task-sharing by mobilizing village-level health workers known as Female Community Health Volunteers (FCHVs). A district-level respondent said:

“*Clients do openly share their problem* [with FCHVs]*… Since they work at community level, they are more aware of peoples who show abnormal activity.*” (District Representative 03, Male).

### Budget allocation and utilization

Most of the national-level respondents said that the proportion of the health budget allocated to mental health has not been publicly stated. In addition, they said that a budget for mental health is allocated only for the mental hospital in Kathmandu and does not go to other hospitals or mental health programs. However, other national-level respondents mentioned that, as part of integrated health care programs, a budget for mental health is allocated to cover the cost of psychiatric wards in regional hospitals. Similarly, a few respondents mentioned that other ministries such as the Ministry of Peace and Reconstruction and the Ministry of Women, Children and Social Welfare are running psychosocial and mental health programs, and said that budgets allocated to these programs have not been accounted for in the total budget allocated to mental health. Still, respondents reported that, even including the budget spent by district and regional health programs and other ministries, the total budget allocated to mental health does not amount to more than 2% of the total health budget. This they found inequitable compared to the disease burden of mental illness in Nepal.

### Supply of psychotropic drugs

Some respondents agreed that there was an insufficient and irregular supply of psychotropic drugs at the district level, which was attributed to insufficient budget and lack of prioritization for mental health. Some district-level respondents believed that other health needs, such as maternal and child health, take precedence over mental health and therefore, the purchase of psychotropic drugs are insufficiently prioritized. A district health worker explained:

“*There is no provision of supplying additional drugs… It is even difficult to supply adequate amount of* [general] *medicine within our budget; in this case, if mental health drug is added, then the situation turns worse.*” (District Representative 01, Male).

Few psychotropic drugs were included in the free drug list, and only two to four drugs were available at the district level. Some district-level respondents criticized that the drugs allocation, coordinated centrally, did not meet the demand. For the effective management of drug supply, respondents recommended that there should be flexible and open policies that allow the districts to purchase the drugs that are needed, even if they are not included in the free drug list.

### Equity and inclusiveness

#### Access to services

Some respondents felt that mental health services were not accessible to all. According to the respondents, services were mostly centered in Kathmandu and some urban areas. In addition, only limited services were available through the government health facilities. Some respondents mentioned that, along with geography and cultural factors, poverty and stigma associated with mental illness are major barriers for access to mental health services. According to these respondents, those who can afford it use modern treatment by traveling far distances, while those who cannot afford to travel ‘outside of the community’ visit traditional healers in their own community. A participant reported,

“*Sakne Raja ko ma janu nasakne deutako ma janu*’ [‘Those who can, they go to the King and those who can’t go to God’]*. While doing the treatment, it is similar thing. Those who can afford, go to medical doctor and those who can’t, go to traditional healer.*” (National Representative 13, Male).

In order to increase access to mental health services in the community, some respondents suggested that the health workers should be provided with training and supervision in mental health and that anti-stigma programs should be implemented in the community. A district-level policymaker said,

“*To reduce stigma and discrimination, community*-*level health workers can be involved because they are directly in touch with the community … They can play a vital role in the rehabilitation process*.” (District Representative 06, Male).

Some respondents thought that involvement of service users was essential to reduce stigma. According to a district-level policymaker, people, who had mental health problems in the past, but are no longer suffering from mental illness, need to be provided with training on mental health and mobilized as trainers:

“*With the known trainer, they develop trust about what s/he just mentioned because they knew s/he had been a sufferer in the past.*” (District Representative 02, Male).

### Ethics

Respondents were asked whether ethics were applied during mental health research and service delivery. Some of them said that there were no mental-health-specific ethical guidelines; however, general health ethics were applied for both mental health research and treatment provisions. For example, for mental health research, ethical approval must be obtained from the Nepal Health Research Council (NHRC) where proposals are evaluated against general health research ethics. Likewise, the MoH, while regulating health service delivery, also monitors whether or not mental health services are provided in an ethical manner. A few respondents said that if complaints of human rights violation of patients arise, then the MoH addresses such issues. A participant mentioned,

“*If any cases regarding violation of rights of the patient comes, then ministry forms a committee to address the issue and based on the report of the committee it takes necessary actions.*” (National Representative 05, Male).

### Mental health information system

The lack of data on the prevalence of mental illness was identified by some respondents as a barrier to prioritizing mental health and allocating necessary resources. A national level representative said:

“*As no research* [at national level] *has been conducted, we do not have background information. So we are not aware of our priority. Government can develop priority area with the help of research of community status.*” (National Representative 08, Male).

Many respondents lacked knowledge about the types of mental health information available from the Health Management Information Systems (HMIS). Those who were aware raised questions about the quality of record and use of the data gathered. A respondent said,

“*I don’t think we have good monitoring system… it is haphazard.*” (National Representative 01, Female). Another participant said, “*case incidences and prevalence are never discussed nor are meetings conducted for discussion.*” (District Representative 03, Male).

Strategies to improve record keeping were suggested by a few of the respondents. They recommended: the development of new mental health indicators similar to the Integrated Management of Childhood Illness (IMCI) indicators and clarity on roles and responsibilities related to record keeping, data analysis and utilization. Another suggestion was to strengthen existing HMIS sections to better manage mental health-related information.

## Discussion

The findings revealed a few enabling factors and many challenges in mental health systems governance in Nepal, as reported by health care policy makers and managers. The enabling policy factors included constitutional provision for health as a human right, inclusion of mental health in the government’s 5-year health plan, provisions for mental health care in the recent National Health Policy, and inclusion of mental health care in the Non-Communicable Disease Multisectoral Action Plan. Other facilitating factors came from increasing participation of NGOs and service user organizations in policy forums, and implementation of mental health care projects in Primary Health Care Centers through government-NGO collaboration.

Policy challenges included non-implementation of mental health policy of 1996, lack of a mental health act despite a fourth revision of the draft, and lack of district-level consultation and participation for mental health policy formulation and planning. Other limiting factors included the inadequate mental health care human resources, inequitable allocation of budget to mental health care, lack of mental health services at the district and primary health care level, inadequate supply of psychotropic drugs, poor mental health care recording systems and insufficient infrastructure for delivery of mental health and psychosocial services. The application of Siddiqi and colleagues’ health systems governance framework has identified the need for: (1) proper implementation of existing policy provisions by assigning dedicated leadership and ensuring governance procedures and mechanisms at the ministry level; (2) greater coordination and collaboration within and outside the health sector through a systems thinking approach; (3) the restructuring of the current health system to better integrate mental health into primary health care; and (4) development and implementation of accountability and transparency measures both at national and district levels. These four domains of needs will be further explained below.

### Implementation of existing mental health policy provisions

Having policies on paper is insufficient; the proper implementation of such policy provisions is needed to ensure access and utilization of mental health services and to reduce the treatment gap. Proper implementation is only possible when governance structures and mechanisms are in place, and there are dedicated people to implement these governance structures. Therefore, good governance is necessary for ensuring effective health care delivery [21] and policy implementation [22].

Good governance is feasible when there is a strong leadership. The absence of such leadership (e.g. through a mental health unit) and lack of clear mental health governance structures in the MoH has prevented the implementation of available provisions in national mental health policy. Greater clarity on mental health governance structures would not only help improve intra-and inter-ministerial coordination but also provide platforms for debate and improvement in mental health provisioning. To achieve this, NGOs and the private sector could play important catalyst and advocacy roles for establishing a central coordination unit for mental health [23]. Many countries affected by conflict and disasters have seized opportunities (during and after the emergency) to make systemic changes in their national mental health systems [24]. The example of Afghanistan shows that NGOs and external development partners played a crucial role in the development of a mental health department there [25].

### Coordination and collaboration

The findings of this study suggest that government institutions work in isolation with regard to mental health care, and coordination with other relevant stakeholders within and outside of MoH is very limited. As there are no or few staff assigned to mental health within the MoH, no one appears to assume responsibility or accountability with regard to mental health care.

The study participants identified that, due to the lack of proper policy direction and guidance from policymakers, there are delays in budget release, which affects the timely training of health workers and the distribution of psychotropic medicines to rural areas. Consequently, patients do not receive medicines on time. Impoverished patients then relapse, and while those who can afford to pay (or borrow) for private practices increase their financial burden. Due to these and many other governance-related factors, the patients and their family members lose confidence in the public health system. To change this situation and regain the trust of patients and their family, there is a greater need for intra-and inter-ministerial coordination and collaboration. As mental health care issues are cross-cutting, we argue for a ‘systematic’ approach for coordination, not only among and across health system building blocks [26] but also beyond the health sector [27], in identifying and addressing barriers to health systems performance in order to improve governance and overall mental health care in Nepal.

### Restructuring the current health system to better integrate mental health

Although the mental health policy and Nepal Health Sector Plan-II promote integration of mental health into primary health care [11], policy provisions have not been applied in practice due to the lack of mental health governance mechanisms at the national as well as the district level. Development of district-level mental health governance is needed to bridge policy and practice. For example, one public health officer from the District Public Health Office could be assigned as a mental health focal person to plan and coordinate all district-level mental health activities. Also, a greater emphasis on community mental health programs can help to deinstitutionalize mental health care [28] and increase awareness among community members, thereby helping to reduce stigma related to mental illness.

As there are relatively few psychiatrists in Nepal, it would not be realistic to deploy them in all 75 districts of Nepal. Therefore, while restructuring the current health systems, Nepal needs to adopt a task-sharing approach [29, 30]. This would mean that prescriptions and medications can be managed by primary health care workers, as suggested by the Mental Health Gap Action Program [31], while identification and referral of people with mental illness can be done by Female Community Health Volunteers (FCHVs) [14].

Despite the challenges related to the overburdening of health volunteers and primary health care workers [32], we argue that integration of mental health into primary health care centers is now feasible, because of the enabling factors that are in place. For instance, existing policies promote integration of mental health into primary health care [11], the government has recently included six psychotropic drugs under the free essential drug list, efforts are under way to develop standardized mental health training manuals, and currently the Health Management Information System (HMIS) has procedures to collect mental health data. The additional cost needed for integration is low and may require only a few weeks of mental health training and quarterly supervisions by psychiatrists.

### Accountability and transparency for better health system performance

The lack of accountability and transparency measures in place, limited access to health services by poor and marginalized groups, and limited engagement and participation of citizens in health affairs are pertinent health governance issues that contribute to low levels of systems effectiveness [11]. Therefore, improved accountability is an important prerequisite for improved health systems performance [33]. However, study findings suggest that concepts such as transparency, accountability and responsiveness are less developed in the field of mental health in Nepal, and there is lack of clarity as to who is responsible for monitoring mental health services and systems. Hence, there is a need for a structure at each health care level that clarifies roles and responsibilities and to ensure transparency and accountability [12]. Likewise, in order to ensure transparency in budget allocation and decision making, we argue that a system of mental health budget planning needs to be instituted and the capacity of existing HMIS needs to be strengthened. Similarly, grievance redressing mechanisms of the Good Governance and Management Act need to be strictly implemented so that the system becomes accountable and responsive toward the needs of the population.

## Strengths and limitations

The results from this study are not easily generalizable to all areas of Nepal; this is because of the presence of a mental health program in the district where interviews were conducted, which is different to many other districts where no mental health services are available. However, the results are indicative of future challenges and solutions that other districts might encounter if mental health programs develop without strengthened governance. Also, in the absence of a mental health policy body at the MoH, and lack of clarity on who are the policymakers for mental health in Nepal, the selection of respondents might have introduced bias. To minimize selection bias, we took a systematic sampling approach in order to represent different stakeholders and institutions potentially involved in mental health policy. Since policymaking is led by government staff, we only included government policymakers currently serving in office. Thus, the results do not capture the views of retired government staff or people from NGOs and the private sector who might have had a significant role in mental health policy making, and we suggest that future studies could assess mental health governance from the perspectives of other stakeholders. One of the limitations of the study is that the inter-rater reliability was not explicitly considered as all the researchers worked on the development, translation, contextualization and pre-testing of the interview checklist. Another limitation of the study is that, due to the small numbers of respondents for the national and district levels, it was not possible to discuss the findings per sub group. As power and prestige play an important role in Nepalese government systems, the power hierarchy between the policy makers and researchers might also have influenced the kind of data being collected and reported.

## Conclusions

The findings of this study suggest that, despite having some facilitating factors, there is a lack of legal provisions for mental health as well as non-implementation of existing policies, due to the absence of leadership at the Ministry of Health and lack of governance mechanisms for mental health. Hence, mental health systems governance in Nepal faces many challenges and needs greater resource inputs and leadership to overcome these challenges. Some positive developments are seen in terms of policy recognition for mental health, as well as participation of NGOs, and some service user groups, in mental health policy and planning. However, many governance principles related to transparency, accountability, ethics, responsiveness and equity are still underdeveloped and need greater attention. Likewise, the lack of trained human resources, frequent relocation of trained health workers, inadequate budget allocation, insufficient infrastructure, poor record keeping, and stigma related to mental health are some of the barriers which need to be addressed through proper governance mechanisms at the national and district level.

## Abbreviations

DOHS: Department of Health Services
DPHO: District Public Health Office
EHCS: Essential Health Care Services
EMERALD: Emerging Mental Health Systems in Low- and Middle-Income Countries
FCHVs: Female Community Health Volunteers
HMIS: Health Management Information Systems
IMCI: Integrated Management of Childhood Illness
MoH: Ministry of Health
NCDs: non-communicable diseases
NGOs: Non-Governmental Organizations
NHRC: Nepal Health Research Council
NHSP: Nepal Health Sector Program
VDC: Village Development Committee
